# Supplementary material for: Detecting the Potential for Consciousness in Unresponsive Patients Using the Perturbational Complexity Index
Source: Brain Sci. 2020 Nov 27;10(12):917. doi: 10.3390/brainsci10120917 (PMC7760168; doi:10.3390/brainsci10120917)
Supplement: Supplementary file 1 [file brainsci-10-00917-s001.pdf]

Supplementary File

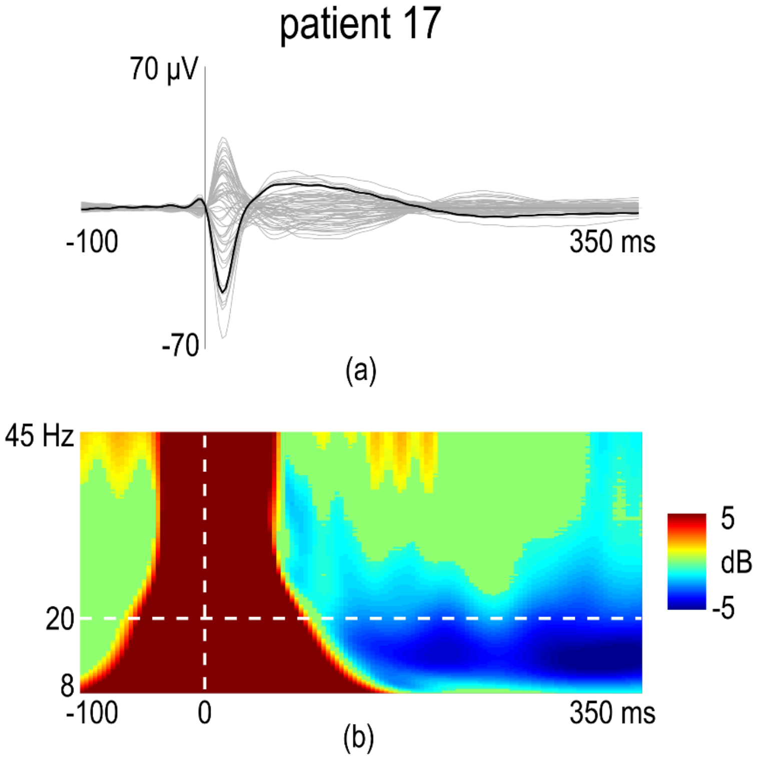

Figure S1. (a) Average transcranial magnetic stimulation (TMS)-evoked potentials (all channels superimposed) recorded in a low-complexity UWS patient. The channel closest to the stimulation site (CP2) is highlighted in black. (b) Event-related spectral perturbation (ERSP) computed for channel CP2 with significance for bootstrap statistics set at $\alpha < 0.05$. Red color indicates a statistically significant power increase, green color indicates absence of any significant modulation of power, blue color represents a significant suppression of power.
